# The Army Canteen

**Published:** 1901-09

**Authors:** 


### The Army Canteen.

No one need be surprised that the effects of the law abolishing the canteen from the United States army are proving disastrous. No man with average worldly sense, who knows that soldiers should be treated like men, and not like school boys, need hesitate to raise his voice in protest. The action of Congress in the first place, was doubtless insincere, for it was largely in response to the agitation of a woman's temperance organization, and was evidently not in accord with the personal conviction of most of the congressmen who voted for it. The attempt to make men total abstainers by act of Congress will fail, as it deserves to fail. The willingness to indulge the impractical ethics of a private organization of agitators at the expense of the comforts and personal rights of the soldiers in the United States army, will evidently soon be rebuked by an enlightened public opinion. During the short time that the law has been in force, it has led to increase in drunkenness and, worse yet, to increase in venereal diseases. Dr. L. L. Seaman, in a paper read before the Association of Military and Naval Surgeons, quoted in the New York Sun, openly declared this to be a fact. The men are obliged to leave the post for the saloon, and find it a short step, evidently, from the saloon to the brothel. The American Medical Association at St. Paul has put itself on record in a resolution, in which it deplores the action of Congress in abolishing the army post exchange, or canteen, and in the interest of discipline, morality and sanitation, recommends its re-establishment at the earliest possible date.

The effect of the act, as we understand it, is to deprive the soldier of the social club, and a part of his social life within the precincts of his post or garrison. If such a law is to stand, we shall all look out, lest every club in civil life be closed ere long by some more of this grandmotherly legislation.—Editorial in the *Philadelphia Medical Journal.*

